# New‐Onset Vitiligo During Dupilumab Treatment: A Clinical Observation and Literature Review

**DOI:** 10.1111/crj.70200

**Published:** 2026-07-09

**Authors:** Xuhui Pu, Suzhen Ju, Yanfang Yu

**Affiliations:** ^1^ Department of Pharmacy Jiading District Central Hospital Affiliated Shanghai University of Medicine and Health Sciences Shanghai China; ^2^ Department of Pulmonary and Critical Care Medicine Jiading District Central Hospital Affiliated Shanghai University of Medicine and Health Sciences Shanghai China

**Keywords:** adverse reactions, atopic dermatitis (AD), chronic lymphocytic leukemia/small lymphocytic lymphoma (CLL/SLL), dupilumab, vitiligo

## Abstract

**Introduction:**

Vitiligo is a rare adverse event associated with dupilumab. This study aims to describe a case of new‐onset vitiligo following dupilumab treatment in a Chinese patient and to systematically review the literature to characterize its clinical features.

**Methods:**

A retrospective case analysis was conducted on a 70‐year‐old male patient who developed vitiligo as a result of dupilumab treatment. Comprehensive clinical, imaging, laboratory, and treatment data were meticulously documented. A systematic literature review revealed a total of 20 cases of dupilumab‐induced vitiligo, which were categorized into two subgroups: improved (*n* = 13) and nonimproved (*n* = 7), based on treatment outcomes. The clinical characteristics of these subgroups were compared using appropriate statistical tests.

**Results:**

The patient developed depigmented patches accompanied by gray hair 4 months after the initiation of dupilumab, with no repigmentation observed despite ongoing treatment. Among the 20 pooled cases, the majority of patients received dupilumab for the treatment of atopic dermatitis (17/20, 85.00%). The onset of vitiligo typically occurred within 3 months of dupilumab initiation (11/18, 61.11%). Most patients presented with new‐onset vitiligo (13/17, 76.47%), and more than half (13/20, 65.00%) exhibited clinical improvement in vitiligo symptoms. Subgroup analysis indicated that the use of topical corticosteroids (*p* = 0.017) was significantly associated with favorable outcomes.

**Conclusion:**

Dupilumab rarely induces new‐onset vitiligo, even after drug discontinuation, potentially through the activation of the Th1/IFN‐γ pathway following IL‐4R blockade. The use of topical corticosteroids may be associated with more favorable outcomes in patients with dupilumab‐induced vitiligo, whereas discontinuing dupilumab might not ensure repigmentation. Clinicians should closely monitor for depigmentation in patients treated with dupilumab, particularly in those with immune dysregulation, as early recognition and the application of topical corticosteroids may improve prognosis.

## Graphical abstract

Dupilumab rarely induces new‐onset vitiligo, even after discontinuation of the drug, potentially through the activation of the Th1/IFN‐γ pathway following IL‐4R blockade. Female sex and the use of topical corticosteroids are associated with improved outcomes, whereas discontinuing dupilumab alone does not ensure repigmentation. Clinicians should closely monitor for depigmentation in patients treated with dupilumab, particularly in those with immune dysregulation, as early recognition and the application of topical corticosteroids may improve prognosis.

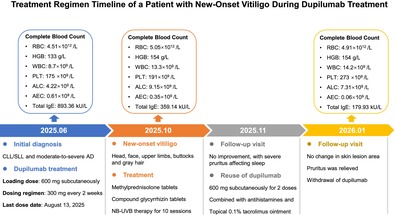

**Abbreviations**
AD: atopic dermatitis
AEC: absolute eosinophil count
ALC: absolute lymphocyte count
CLL/SLL: chronic lymphocytic leukemia/small lymphocytic lymphoma
EASI: eczema area and severity index
HGB: hemoglobin
IFN‐γ: interferon‐γ
NB‐UVB: narrow‐band ultraviolet B
PLT: platelet
RBC: red blood cell
RCM: reflectance confocal microscopy
TCI: topical calcineurin inhibitor
TCS: topical corticosteroid
TNF: tumor necrosis factor
WBC: white blood cell

## 1 Introduction

Dupilumab is a fully human IgG4 monoclonal antibody that specifically binds to the interleukin (IL)–4 receptor α subunit within the IL‐4 and IL‐13 receptor complex. This binding inhibits IL‐4 and IL‐13 signaling, subsequently alleviating inflammatory responses. This agent has been widely utilized to treat Type 2 inflammatory diseases that are inadequately controlled by conventional therapies, such as atopic dermatitis (AD) and bronchial asthma, demonstrating favorable efficacy and a well‐established safety profile. Common adverse reactions compared to placebo include injection site reactions, conjunctivitis, and respiratory tract infections [1, 2]. However, with the increasing clinical use of dupilumab, rare adverse events have been gradually reported. A case–control study based on the VigiBase database in 2020 [3] identified nine cases of vitiligo associated with dupilumab use (IC_025_ = 0.3). Nonetheless, reports of dupilumab‐induced vitiligo‐like changes in the Chinese population remain limited.

Previous studies have demonstrated that the disruption of the tumor microenvironment is a key pathogenic mechanism in patients with chronic lymphocytic leukemia/small lymphocytic lymphoma (CLL/SLL) [4]. Notably, exhausted CD8^+^ T cells can be detected even in patients with early‐stage CLL [5]. In contrast, vitiligo is currently classified as a CD8^+^ T cell‐mediated autoimmune disease [6], indicating that CD8^+^ T cells may have opposing roles in these two conditions. However, an epidemiological study on vitiligo [7] found no significant association between CLL and an increased incidence of vitiligo. To date, reports of vitiligo following dupilumab treatment in patients with AD and concurrent chronic hematologic diseases remain extremely rare.

Here, we present a case of new‐onset vitiligo in a Chinese patient with AD and CLL/SLL that emerged following the administration of dupilumab. Additionally, we conducted a comprehensive literature review and performed a subgroup analysis of the patients' clinical characteristics based on varying treatment outcomes associated with dupilumab‐induced vitiligo, aiming to enhance the understanding of this rare adverse drug reaction and its management strategies.

## 2 Methods

### 2.1 Case Selection

The patient diagnosed with AD and CLL/SLL was retrospectively identified at the Jiading District Central Hospital Affiliated Shanghai University of Medicine & Health Sciences. Clinical data were extracted from electronic medical records, with the last follow‐up conducted on January 31, 2026. This study received approval from the ethics committee of the Jiading District Central Hospital (No. 20260155), and written informed consent was obtained from the patient for the publication of this article and accompanying images.

### 2.2 Images Taken

Images of the patient's skin lesions were obtained through Wood's lamp examination. Reflectance confocal microscopy (RCM) images were captured using a VivaScope 1500 (Caliber I.D., Andover, MA, United States).

### 2.3 Statistical Analysis

Statistical analyses were conducted using SPSS 27.0 software (SPSS Inc., Chicago, IL, United States). Normally distributed measurement data were presented as mean ± standard deviation (SD), whereas nonnormally distributed data were expressed as median (M) and interquartile range (P_25_ and P_75_). Intergroup comparisons were performed using *t* tests and Mann–Whitney *U* tests, respectively. Categorical data were presented as counts and percentages (%), and intergroup differences were analyzed using *χ* ^2^ tests or Fisher's exact probability method. All comparisons were two‐sided tests, with *p* < 0.05 considered statistically significant.

## 3 Results

### 3.1 Case Presentation

**FIGURE 1:**
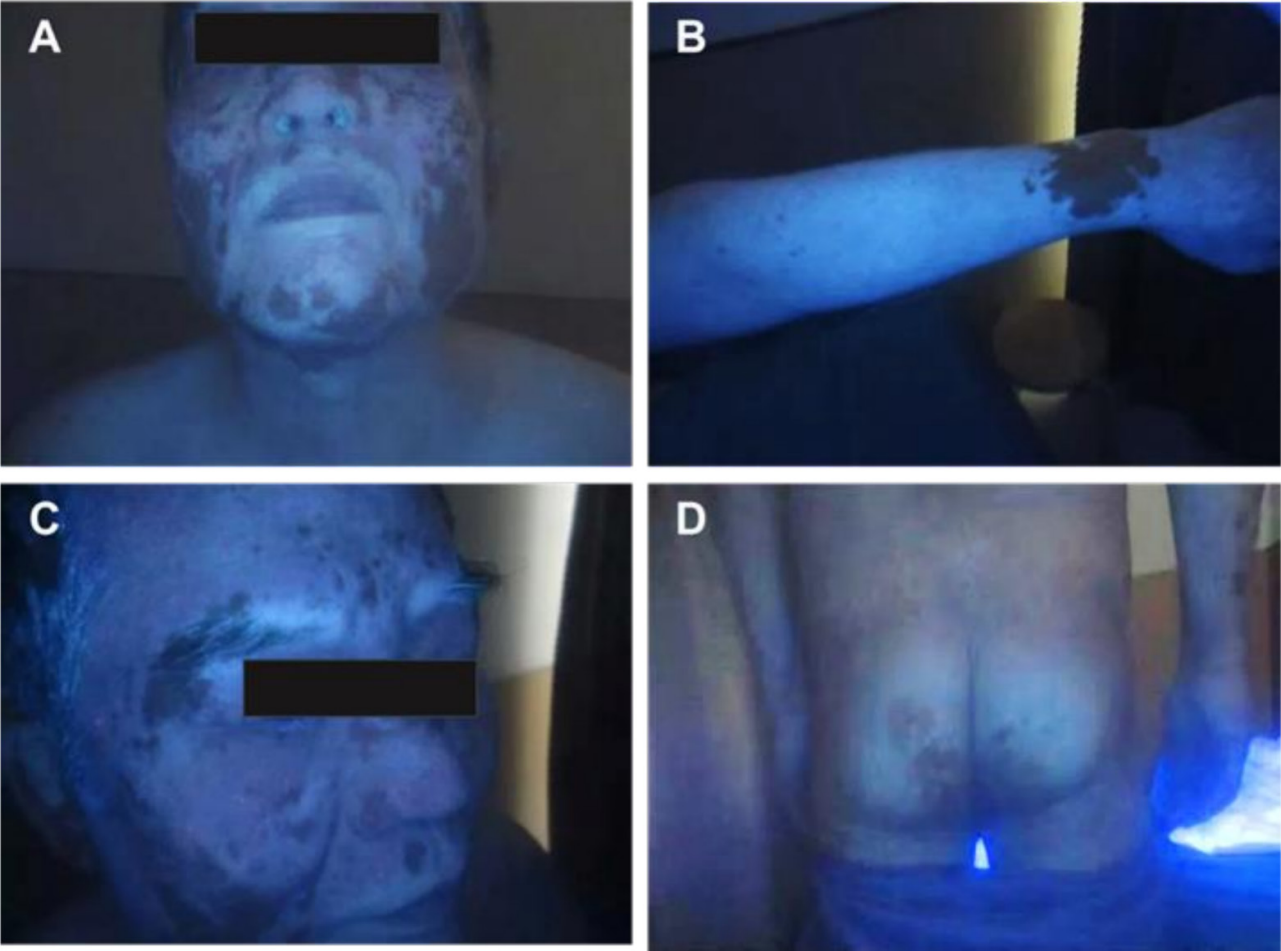
Wood's lamp examination of depigmented patches. (A) Head and face; (B) upper limbs; (C) gray hair; and (D) buttocks.

The patient was a 70‐year‐old male weighing 83 kg, who presented with a generalized rash accompanied by pruritus for 4 months. He visited the Dermatology Department of Jiading District Central Hospital on June 18, 2025. The patient developed maculopapular eruptions on his face in February 2025, initially without pruritus. Following treatment with antihistamines and topical corticosteroids, the rash worsened and gradually spread to his entire body, coinciding with the onset of pruritus, which prompted his return visit. The patient had a 14‐year history of asthma, regularly using an indacaterol inhaler for long‐term control with stable condition. He was diagnosed with chronic lymphocytic leukemia/small lymphocytic lymphoma (CLL/SLL) 1 year ago and is currently under a watch‐and‐wait strategy. He denied any history of food or drug allergies, as well as any personal or family history of vitiligo. Physical examination revealed symmetrical erythema, papules, scales, scratch marks, and hyperpigmentation throughout the body, accompanied by dry and desquamating skin. Complete blood count results indicated red blood cells 4.51 × 10^12^/L, hemoglobin 133 g/L, white blood cells 8.7 × 10^9^/L, platelets 175 × 10^9^/L, absolute lymphocyte count 4.22 × 10^9^/L, absolute eosinophil count 0.61 × 10^9^/L, and total IgE 893.36 kU/L. Ultrasound revealed the largest superficial lymph node in the right axilla measuring 44 × 14 mm. The Eczema Area and Severity Index (EASI) score was 48 points, leading to a diagnosis of moderate‐to‐severe AD. The initial dose of dupilumab, 600 mg, was administered subcutaneously on June 18, 2025, followed by a maintenance therapy regimen of 300 mg every 2 weeks. After two treatments, the rash showed slight improvement, as indicated by a decrease in the EASI score to 28 points. However, no further improvement was observed despite the addition of antihistamine therapy. The patient subsequently requested the discontinuation of dupilumab treatment after the last dose on August 13, 2025. On October 22, 2025, approximately 4 months after the initiation of dupilumab and 2 months following its discontinuation, the patient developed multiple vitiligo‐like depigmented patches on the head, face, bilateral upper limbs, and buttocks, accompanied by gray hair (Figure 1).

**FIGURE 2:**
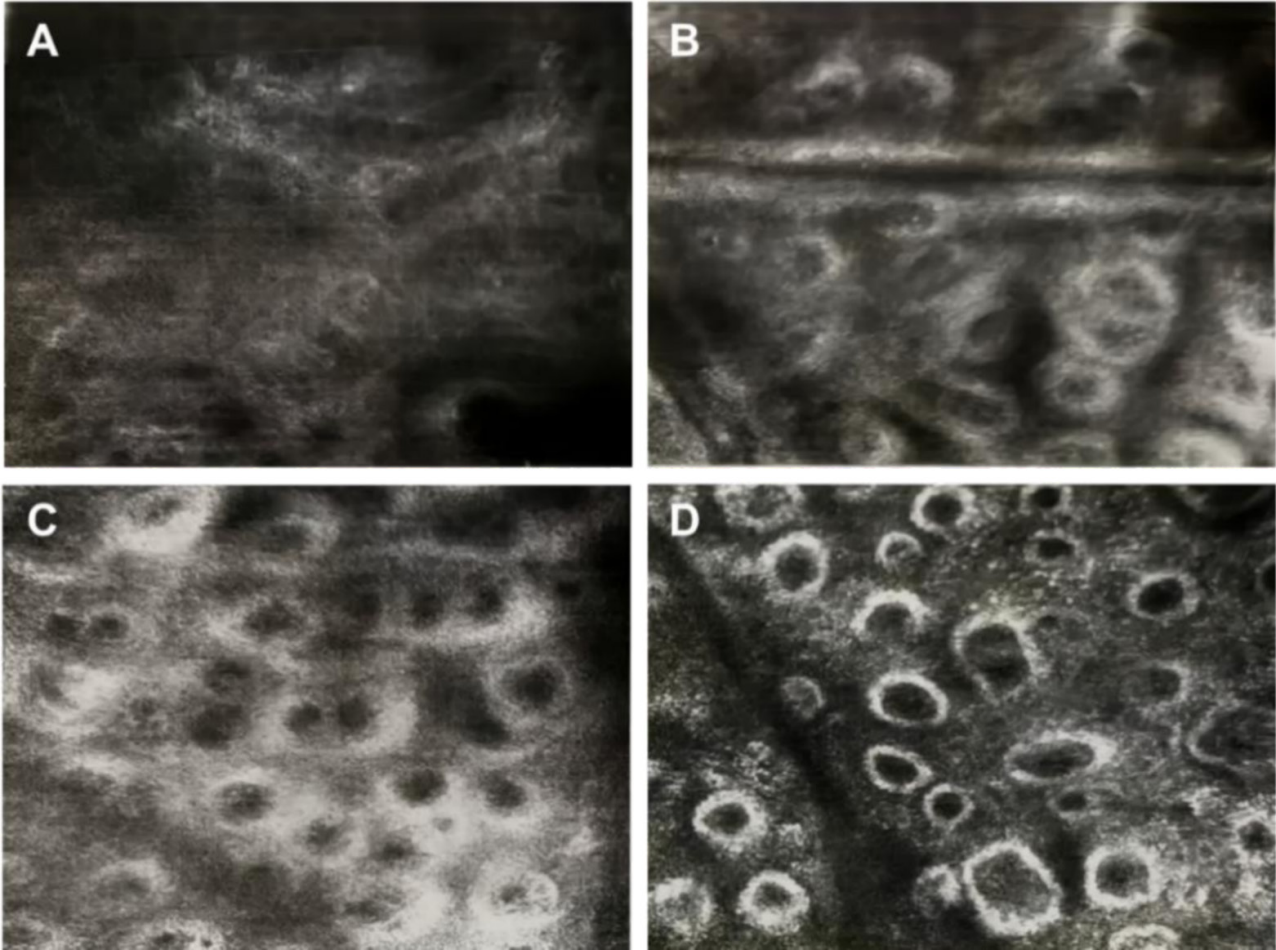
Reflectance confocal microscopy (RCM) examination of the patient's skin. (A, B) White patches on the face and left hand with marked melanocyte absence in the basal layer and indistinct melanin rings; (C) semicircular melanin rings at the white patch border; and (D) intact and clear annular melanin rings in normal skin.

Laboratory tests showed red blood cells 5.05 × 10^12^/L, hemoglobin 154 g/L, white blood cells 13.3 × 10^9^/L, platelets 191 × 10^9^/L, absolute lymphocyte count 9.15 × 10^9^/L, absolute eosinophil count 0.35 × 10^9^/L, total IgE 359.14kU/L, IL‐10 3.8 pg/mL, TNF‐α 0 pg/mL, TNF‐β 2.77 pg/mL, and IFN‐γ 1.83 pg/mL. The indices related to rheumatology, immunology, and thyroid function tests were all within normal limits. Two days later, lymphocyte subset analysis revealed a total lymphocyte absolute count of 12 676/μL, with CD4^+^ T lymphocytes at 617/μL, CD8^+^ T lymphocytes at 301/μL, B lymphocytes at 31/μL, and NK cells at 878/μL. RCM examination indicated that vitiligo was in the progressive stage (Figure 2). Subsequently, the patient was prescribed oral methylprednisolone tablets, compound glycyrrhizin tablets, and vitamin D, in addition to undergoing 10 sessions of narrow‐band ultraviolet B (NB‐UVB) therapy.

**FIGURE 3:**
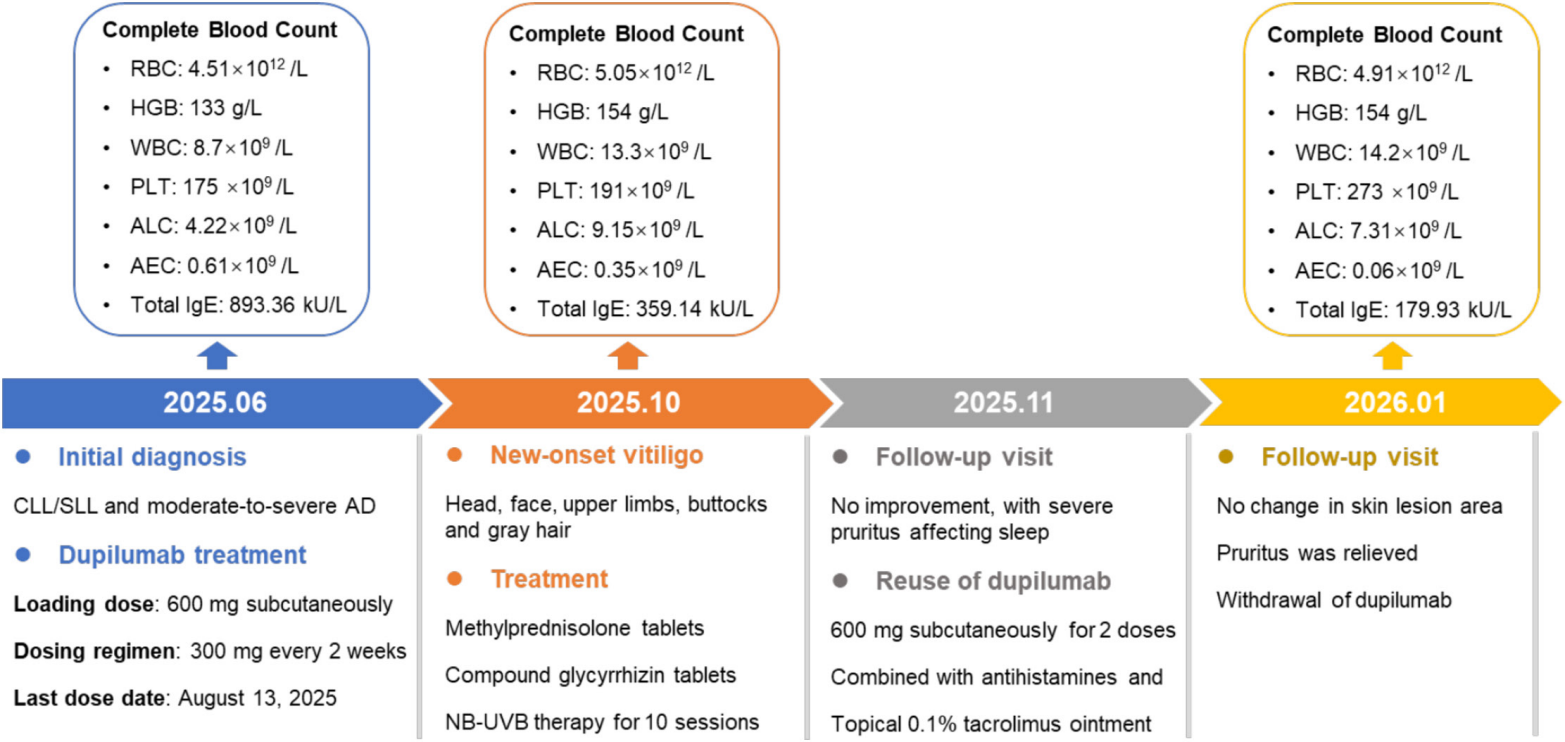
Timeline of the patient's treatment regimen. CLL/SLL, chronic lymphocytic leukemia/small lymphocytic lymphoma; AD, atopic dermatitis; AEC, absolute eosinophil count (range: 0.02–0.52 × 10^9^/L); ALC, absolute lymphocyte count (range: 1.1–3.2 × 10^9^/L); HGB, hemoglobin (range: 115–150 g/L); PLT, platelet (range: 125–350 × 10^9^/L); RBC, red blood cell (range: 3.8–5.1 × 10^12^/L); WBC, white blood cell (range: 3.5–9.5 × 10^9^/L).

In November 2025, the patient returned for a follow‐up visit. The depigmentation of the skin showed no improvement, and the severe pruritus negatively impacted his sleep quality. The patient requested to continue dupilumab treatment (600‐mg subcutaneous injection, two doses in total) in combination with antihistamines and topical 0.1% tacrolimus ointment. By January 2026, during another follow‐up, the extent of skin lesions remained largely unchanged; however, the pruritus symptoms had somewhat alleviated. Complete blood count results showed red blood cell count 4.91 × 10^12^/L, hemoglobin 154 g/L, platelets 273 × 10^9^/L, white blood cells 14.2 × 10^9^/L, absolute lymphocyte count 7.31 × 10^9^/L, absolute eosinophil count 0.06 × 10^9^/L, and total IgE level 179.93kU/L. Ultrasound examination revealed enlargement of peripheral superficial lymph nodes, with the largest node located in the right axilla (46 × 15 mm). The patient declined to undergo histopathological examination and discontinued dupilumab treatment, with the last follow‐up visit occurring on January 31, 2026. The entire treatment timeline of the patient is illustrated in Figure 3.

### 3.2 Literature Review

To date, a total of 20 patients with dupilumab‐induced vitiligo have been reported [8, 9, 10, 11, 12, 13, 14, 15, 16, 17], including the case presented in this study. The clinical characteristics of these patients are summarized in Table 1.

**TABLE 1:** Clinical characteristics of dupilumab‐induced vitiligo patients reported in the literature.

Year, author	Patient	Age	Gender	Dupilumab indication	Previous history of vitiligo	Interval from dupilumab initiation to vitiligo onset (months)	Distribution of vitiligo	Dupilumab cessation	Treatment of vitiligo	Vitiligo outcome
2025, Bazzacco et al. [8]	1	57	F	Severe asthma	No	1.5	Perilabial	Yes	Topical tacrolimus 0.1% and mometasone	Complete remission after 4 months
2024, Sainan et al. [9]	2	10	F	Severe AD	No	10	Both upper limbs	Yes	Topical mometasone and tacrolimus ointment	Repigmentation at 4 months of follow‐up
2024, Shao et al. [10]	3	80	M	Moderate AD	No	2	Chest, back, and both lower limbs	Yes	Glycyrrhizin	Stability
	4	14	F	Moderate AD	No	1	Forehead and gray hair	No	Topical hydrocortisone butyrate ointment	Partial improvement after 5 months
2024, Ferreirinha et al. [11]	5	30	F	AD	No	24	Popliteal, cubital, and axillary flexures	No	Tacrolimus 0.1% ointment and NB‐UVB	Partial repigmentation after 6 months
	6	20	M	AD	No	3	Wrists and perimalleolar	Yes	Tacrolimus 0.1% ointment	Stability
2023, Napolitano et al. [12]	7	52 ± 38.18^a^	M	AD	N/A	1.97 ± 2.67^a^	N/A	No	Topical corticosteroids and NB‐UVB	Resolution
	8	52 ± 38.18^a^	F	AD	N/A	1.97 ± 2.67^a^	N/A	No	Topical corticosteroids and NB‐UVB	Resolution
2023, Picone et al. [13]	9	79	M	Severe AD	No	1	Scalp, neck, and back of the hands	No	Topical corticosteroids and NB‐UVB	Complete remission at 4 months of follow‐up
2023, Ren et al. [14]	10	18	M	AD	No	2	Bilateral cheeks, neck	Yes	Tacrolimus 0.1% ointment	Ongoing
	11	50	F	AD	No	6	Right hairline, neck and chest	Yes	Mometasone 0.1% ointment and NB‐UVB	Resolution
	12	40	F	AD	No	5	Hands and feet	No	None	Resolution
	13	50	M	AD	No	3	Hands, legs and feet	No	Triamcinolone acetonide 0.1% ointment and tacrolimus 0.1%	Resolution
	14	18	F	AD	Yes	4	Pelvis, left leg and lower torso	No	Triamcinolone acetonide 0.1% ointment	Resolution
	15	43	F	NP	Yes	1	Face, bilateral breasts, chest, abdomen, bilateral forearms and proximal thighs	No	Betamethasone dipropionate 0.05% cream, tacrolimus 0.1% ointment and NB‐UVB	Stability
	16	52	M	NP	Yes	3	Forehead, glabella, nose and cheeks	No	Ruxolitinib 1.5% cream and NB‐UVB	Stability
2023, Muradova et al. [15]	17	71	F	AD	No	2	Face, neck, chest and arms	N/A	Topical ruxolitinib	Partial improvement
2022, Xin et al. [16]	18	72	M	AD	N/A	10	Right forehead	No	None	Stability
2021, Takeoka et al. [17]	19	17	M	Severe AD	Yes	3	Forehead and grey hair	Yes	Hydrocortisone butyrate, delgocitinib ointment and excimer light therapy	Slight repigmentation but its area has not shrunk after 17 months
2026, Xuhui et al.	20	70	M	Moderate to severe AD	No	4	Head, face, upper limbs, buttocks and gray hair	Yes	Oral methylprednisolone tablets, glycyrrhizin, topical 0.1% tacrolimus ointment, and NB‐UVB	Stability

Abbreviations: AD, atopic dermatitis; F, female; M, male; N/A, not available; NB‐UVB, narrow‐band ultraviolet B; NP, nasal polyposis.

^a^ Data presented as mean ± SD from Napolitano et al. [12].

### 3.3 Clinical Characteristics Analysis by Treatment Outcome

Based on treatment outcomes, 20 patients with dupilumab‐induced vitiligo were classified into improved (*n* = 13) and nonimproved (*n* = 7) subgroups. The clinical characteristics of the two cohorts are presented in Table 2. No significant differences were observed in age and time to vitiligo onset between the two groups (*p* = 0.442 and *p* = 0.592, respectively). A trend toward a higher proportion of females in the improved group (69.2% vs. 14.3%) and males in the nonimproved group (85.7% vs. 30.8%) was noted (*p* = 0.057). Regarding dupilumab discontinuation, 30.8% (4/13) of patients in the improved group ceased treatment, compared to 57.1% (4/7) in the nonimproved group; no statistically significant difference was observed (*p* = 0.377). In terms of treatment modalities, the use of topical corticosteroids (TCS) was significantly more prevalent in the improved group than in the nonimproved group (76.9% vs. 14.3%, *p* = 0.017). In contrast, the usage of topical calcineurin inhibitors (TCI) (30.8% vs. 57.1%, *p* = 0.356) and NB‐UVB (38.5% vs. 42.9%, *p* = 1.000) did not differ significantly between the two groups.

**TABLE 2:** Comparison of clinical characteristics by treatment outcome in dupilumab‐induced vitiligo patients.

Characteristics	Improved group (*n* = 13)	Nonimproved group (*n* = 7)	*p* value
Age (years)	41.54 ± 24.79	50.71 ± 25.03	0.442
Gender			0.057
Female	9 (69.2%)	1 (14.3%)	
Male	4 (30.8%)	6 (85.7%)	
Time interval to onset of vitiligo (months)	4.96 ± 6.29	3.57 ± 2.99	0.592
Dupilumab cessation			0.377
Yes	4 (30.8%)	4 (57.1%)	
No	8 (61.5%)	3 (42.9%)	
Treatment			
TCS	10 (76.9%)	1 (14.3%)	**0.017**
TCI	4 (30.8%)	4 (57.1%)	0.356
NB‐UVB	5 (38.5%)	3 (42.9%)	1.000

*Note:* The bold text indicates the statistically significant difference between the two groups (*p* < 0.05).

Abbreviations: NB‐UVB, narrow‐band ultraviolet B; TCI, topical calcineurin inhibitor; TCS, topical corticosteroid.

## 4 Discussion

The patient in this study presented with AD complicated by CLL/SLL. Prior to the initiation of dupilumab treatment, diagnostic evaluations—including complete blood count, superficial lymph node palpation, and abdominal ultrasound—confirmed asymptomatic early‐stage CLL/SLL, for which no pharmacological treatment was administered. The patient received dupilumab treatment for 2 months (totaling five injections) following the recommended dosage in the prescribing information but discontinued therapy due to an inadequate response. Two months after treatment cessation (i.e., 4 months after the first dupilumab administration), the patient developed generalized multiple depigmented lesions accompanied by gray hair. Vitiligo was diagnosed by RCM examination, and the patient received approximately 1 month of treatment, which included methylprednisolone, compound glycyrrhizin, and NB‐UVB therapy. After treatment, no repigmentation or reduction was observed in the depigmented areas. Due to aggravated pruritus, the patient requested subcutaneous injections of dupilumab, which were administered once on November 19, 2025, and again on December 3, 2025, in combination with ebastine and tacrolimus ointment. Following this treatment, the pruritus symptoms alleviated, and the depigmented areas did not further expand. Given the patient's concomitant CLL, a follow‐up complete blood count after the onset of vitiligo revealed a marked increase in the absolute T‐lymphocyte count. However, the volumes of superficial lymph nodes, liver, and spleen showed no significant enlargement compared to pretreatment status, and there were no symptoms such as fever, infection, or fatigue. Existing studies have confirmed the safety of dupilumab in patients with hematologic disorders [18]. After evaluation by a hematologist, initiation of CLL treatment was deemed unnecessary at that time [4]. Although vitiligo occurred 2 months after the discontinuation of the drug, calculated residual plasma concentrations of dupilumab remained above the lower limit of detection according to the literature [19], suggesting that the new‐onset vitiligo was associated with dupilumab use. Assessment using the Naranjo scale yielded a score of 3 [20], indicating a “possible” causal relationship between dupilumab and the occurrence of vitiligo.

A study based on the FAERS database revealed that dupilumab had the highest incidence of vitiligo (70/86 cases, 81.40%) and the strongest association (ROR = 2.67, 95% CI 2.11–3.40; IC_025_ = 1.09) among Type 2 immune response inhibitors [21]. Among these cases, 14.29% (10/70) of patients experienced severe adverse reactions, with no reported fatalities. The median time to onset of vitiligo‐related adverse reactions was 95 days (IQR 18.5–121), and patients with severe AD were more susceptible than those with mild‐to‐moderate AD. At the time of the study, 20 dupilumab‐induced vitiligo cases had been reported in the literature, including the present case [8, 9, 10, 11, 12, 13, 14, 15, 16, 17], as detailed in Table 1. Geographically, the United States (8/20, 40.00%) and Europe (6/20, 30.00%) represented the highest proportions of cases, potentially due to the earlier approval and longer duration of dupilumab use in these regions. The age of patients ranged from 10 to 80 years, with a male‐to‐female ratio of 1:1. Notably, the incidence of vitiligo was higher among AD patients (17/20, 85.00%), consistent with findings from studies utilizing the VigiBase database [3]. Among the 20 patients, available data indicate that the majority of cases (13/17, 76.47%) presented with new‐onset vitiligo. Vitiligo typically occurred within 3 months after dupilumab initiation (11/18, 61.11%), with a median time of 3.0 months, slightly earlier than the findings reported in the FAERS database study [21]. Depigmentation most frequently involved the head and neck region (11/18, 61.11%). Over half of the patients (13/20, 65.00%) exhibited improvement in vitiligo symptoms. Among these 13 improved patients, 10 had received TCS treatment (10/13, 76.90%). The shortest time to achieve repigmentation (symptom relief) was 4 months.

Subgroup analysis revealed a trend toward a higher proportion of females in the improved group and a significant association between the use of TCS and favorable treatment outcomes. Conversely, factors such as age, time to vitiligo onset, dupilumab discontinuation, use of TCI, and NB‐UVB therapy did not differ between the improved and nonimproved groups. Current evidence regarding gender differences in the efficacy of conventional vitiligo treatments is limited, as most studies lack gender‐stratified analyses, resulting in no clear consensus [22]. The question of whether female patients demonstrate better responsiveness to pharmacological treatments for vitiligo remains unresolved, necessitating further in‐depth studies to validate this observation. The utilization rate of TCS in the improved group was significantly higher (76.9% vs. 14.3%, *p* = 0.017), indicating that TCS may serve as an effective first‐line treatment option for dupilumab‐related vitiligo, aligning with the standard treatment regimen for vitiligo [23]. The lack of significant differences in the use of TCI and NB‐UVB may be due to the small sample size or variations in treatment protocols. Larger prospective studies are needed to clarify the role of these therapeutic modalities in this specific clinical context. Notably, the nonimproved group had a higher rate of dupilumab cessation than the improved group (57.1% vs. 30.8%). Although this difference was not statistically significant, it highlights that discontinuing dupilumab may not ensure repigmentation and that active treatment for vitiligo remains necessary. The patient in this study developed depigmentation 2 months after cessation of dupilumab injections, and no progression of vitiligo was observed upon readministration of dupilumab, suggesting that dupilumab‐induced vitiligo may not be directly dose‐dependent. It should be noted that subgroup analyses in this study were limited by the small number of reported cases and the heterogeneity of available data. Therefore, conclusions regarding prognostic factors and potential pathogenic mechanisms are exploratory and hypothesis‐generating, and require further validation.

AD, asthma, chronic rhinosinusitis with nasal polyposis, and prurigo nodularis are primarily driven by Th2‐type inflammatory responses. Dupilumab exerts its therapeutic effects in these diseases by blocking the IL‐4/IL‐13 signaling pathways, thereby suppressing Th2‐type immune activity [24]. In contrast, vitiligo is characterized as a classic Th1/cytotoxic T cell‐mediated autoimmune disease, where the core pathogenesis involves the destruction of melanocytes by IFN‐γ and CD8^+^ T cells [25]. CD8^+^CD49a^+^ tissue‐resident memory T (Trm) cells are localized in the basal epidermis, adjacent to the collagen IV‐rich basement membrane. In a healthy state, these cells remain quiescent and, once formed, can persist long‐term in tissues without relying on the replenishment of circulating T cells. During dupilumab therapy, Th2 immunity is suppressed, potentially shifting the immune balance toward a Th1‐dominant response. CD49a^+^ CD8^+^ Trm cells accumulate extensively due to the loss of antagonism by Th2 cytokines, transitioning from a quiescent state to an effector state. These cells produce IFN‐γ and rapidly upregulate the expression of perforin and granzyme B upon IL‐15 stimulation, thereby promoting a robust cytotoxic response that targets melanocytes and induces vitiligo [26]. Even after the cessation of dupilumab, these cells do not spontaneously resolve; instead, they persist as “local immune memory,” continuing to eliminate repopulating melanocytes [27]. Consequently, after drug discontinuation, vitiligo neither progresses rapidly nor achieves spontaneous repigmentation. Similar phenomena have also been reported in anti‐PD‐1/PD‐L1 immunotherapy, where immune checkpoint inhibitors can induce or exacerbate vitiligo, often persisting after treatment withdrawal [28]. Although the mechanism by which dupilumab induces vitiligo differs from that of immune checkpoint inhibitors, the ultimate outcome remains similar: Once the drug triggers the clonal expansion and functional maturation of Trm cells, these cells acquire “autonomy” and are not reversed by drug discontinuation. This serves as a reminder to clinicians that when vitiligo‐like manifestations occur during dupilumab therapy, merely discontinuing the drug and observing is insufficient. Instead, early assessment, active treatment, enhanced psychological support, and long‐term follow‐up should be implemented.

Brunner et al. [29] reported that immune dysregulation in vitiligo patients involves not only the Th1/IFN‐γ pathway but also multiaxis polarized immune disorders. Studies have demonstrated upregulated expression levels of Th2‐related molecules, such as IL‐4R and the anti‐inflammatory factor IL‐10, in nonlesional skin, suggesting that the Th2 axis may play a role in immune regulation during the early or subclinical stages of vitiligo pathogenesis. It is widely accepted that IL‐10 can inhibit Th1‐mediated melanocyte damage. However, the role of IL‐4R in the pathogenesis of vitiligo is more complex. On one hand, it is believed to participate in the feedback regulation mechanism prior to Th1 activation, thereby inhibiting immune imbalance in the Th1 pathway. On the other hand, it is thought to inhibit melanin production by melanocytes, leading to depigmentation. In this context, lymphocyte subset analysis revealed a significant increase in the absolute total lymphocyte count, with detectable levels of IFN‐γ. Considering the patient's history of CLL/SLL, it is hypothesized that the inhibition of IL‐4R by dupilumab may enhance Th1 pathway activity. Notably, no TNF‐α was detected in the patient's blood, and IL‐10 levels were within normal ranges, suggesting that the occurrence of vitiligo may not depend on the TNF‐α pathway but is primarily mediated through the IFN‐γ‐CXCL9/10‐CXCR3 axis [30]. However, the specific mechanisms require further investigation for elucidation.

## 5 Conclusions

Dupilumab can rarely induce new‐onset vitiligo, as demonstrated in this case involving a patient with AD and concurrent CLL/SLL. The onset of vitiligo occurred even after the discontinuation of dupilumab, likely due to the activation of the Th1/IFN‐γ pathway following IL‐4R blockade. The use of TCS may be associated with more favorable outcomes in patients with dupilumab‐induced vitiligo, whereas discontinuing dupilumab might not ensure repigmentation. Clinicians should remain vigilant for depigmentation in patients treated with dupilumab, particularly those with underlying immune dysregulation. Prompt recognition, combined with the application of TCS, may improve prognosis.

## Author Contributions

X.P. contributed to the study design, data collection, and manuscript drafting. S.J. conducted data interpretation, literature review, and statistical analyses. Y.Y. supervised the process and revised the manuscript. All authors reviewed and approved the final manuscript for submission.

## Funding

This work was supported by the Clinical Specialty Capacity Enhancement Training Program of Jiading District Central Hospital (JZXLCZK‐2024‐06).

## Ethics Statement

This study was approved by the Ethics Committee of Jiading District Central Hospital (No. 20260155). Written informed consent was obtained from the patient in this study.

## Conflicts of Interest

The authors declare no conflicts of interest.

## Data Availability

The data that support the findings of this study can be obtained from the corresponding author upon reasonable request.
